# Assessing Causality Between Plasma Brain‐Derived Neurotrophic Factor With Major Depression Disorder: A Bidirectional Mendelian Randomization Study

**DOI:** 10.1002/brb3.70425

**Published:** 2025-03-18

**Authors:** Ming Chen, Hao‐Zhang Huang, Yi‐Hui Liu, Qiang Li, Lin‐Yan Fu, Cai‐Lan Hou

**Affiliations:** ^1^ Guangdong Mental Health Center, Guangdong Provincial People's Hospital (Guangdong Academy of Medical Sciences) Southern Medical University Guangzhou China; ^2^ Department of Cardiology, Guangdong Provincial People's Hospital (Guangdong Academy of Medical Sciences) Southern Medical University Guangzhou China; ^3^ Interventional Center of Valvular Heart Disease, Beijing Anzhen Hospital Capital Medical University Beijing China

**Keywords:** brain‐derived neurotrophic factor, major depression disorder, mendelian randomization study

## Abstract

**Purpose:**

This study employed a two‐sample Mendelian randomization (MR) approach to investigate the bidirectional relationship between brain‐derived neurotrophic factor (BDNF) and major depressive disorder (MDD), addressing gaps left by prior observational studies.

**Methods:**

We utilized Genome‐Wide Association Study (GWAS) datasets, including MDD information from the Psychiatric Genomics Consortium (PGC) and the UK Biobank (*N* = 500,199), along with plasma BDNF measurements from the FinnGen Consortium (*N* = 619). In a subsequent phase, we analyzed MDD data from FinnGen (*N* = 448,069) with plasma BDNF data from three additional GWAS sources: UK Biobank (*N* = 33,924), deCODE (*N* = 35,353), and INTERVAL (*N* = 3301). Multiple MR methods were applied to ensure a robust analysis.

**Results:**

The inverse variance weighted (IVW) method revealed no significant association between plasma BDNF levels and the risk of developing MDD (IVW odds ratio [OR] = 1.00, 95% confidence interval [CI] = 0.99–1.01, *p* = 0.769). Similarly, no causal effect of the *BDNF* gene on MDD was identified (OR = 0.91, CI = 0.23–3.56, *p* = 0.893). Furthermore, there was no evidence supporting a causal link between MDD and plasma BDNF levels (OR = 0.99, CI = 0.89–1.09, *p* = 0.783). The second phase of analysis confirmed the absence of bidirectional causal relationships.

**Conclusion:**

This bidirectional MR analysis provides no evidence of a causal association between plasma BDNF levels and MDD. These findings prompt a re‐evaluation of plasma BDNF as a biomarker for MDD and emphasize the need for further investigation into its functional role within the plasma as well as its levels and activity in the brain and cerebrospinal fluid.

## Graphical abstract

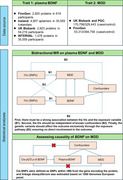

## 1 Introduction

Major depressive disorder (MDD) is a leading cause of global disease burden, affecting over 300 million people worldwide (Ferrari et al. 2013). Brain‐derived neurotrophic factor (BDNF) is essential for the survival, growth, and maintenance of neurons in brain circuits that regulate emotional and cognitive functions (Phillips 2017).

Emerging evidence links reduced levels of both central and plasma BDNF with various neuropsychiatric disorders, suggesting its potential as a biomarker for underlying pathologies (Bazzari and Bazzari 2022; Domitrovic Spudic et al. 2022). Previous studies indicate that decreased plasma BDNF levels are strong predictors of MDD occurrence (Liu et al. 2022; Polyakova et al. 2015; Cattaneo et al. 2016). However, despite evidence of a relationship between plasma BDNF and MDD, factors such as heterogeneous results, small sample sizes, publication bias, and differences in BDNF measurements (serum vs. plasma) complicate interpretation.

Mendelian randomization (MR) has been utilized in psychiatric disorder research, including MDD, to elucidate etiological mechanisms and improve our understanding of treatment strategies (Liu et al. 2024; X. Zhang, Q. Lu, et al. 2024). Although genetic studies have linked depressive behavior with altered BDNF functioning (Dwivedi et al. 2003, 2009), the causal relationship between plasma BDNF and MDD remains unclear. MR allows for the examination of potential causal associations between exposures and outcomes. Consequently, this study conducted a bidirectional MR analysis to investigate whether genetic variability in individuals with MDD is causally associated with plasma BDNF levels. Understanding this bidirectional relationship is crucial for grasping the complex interactions underlying these disorders.

## 2 Method

### 2.1 Study Design

**FIGURE 1:**
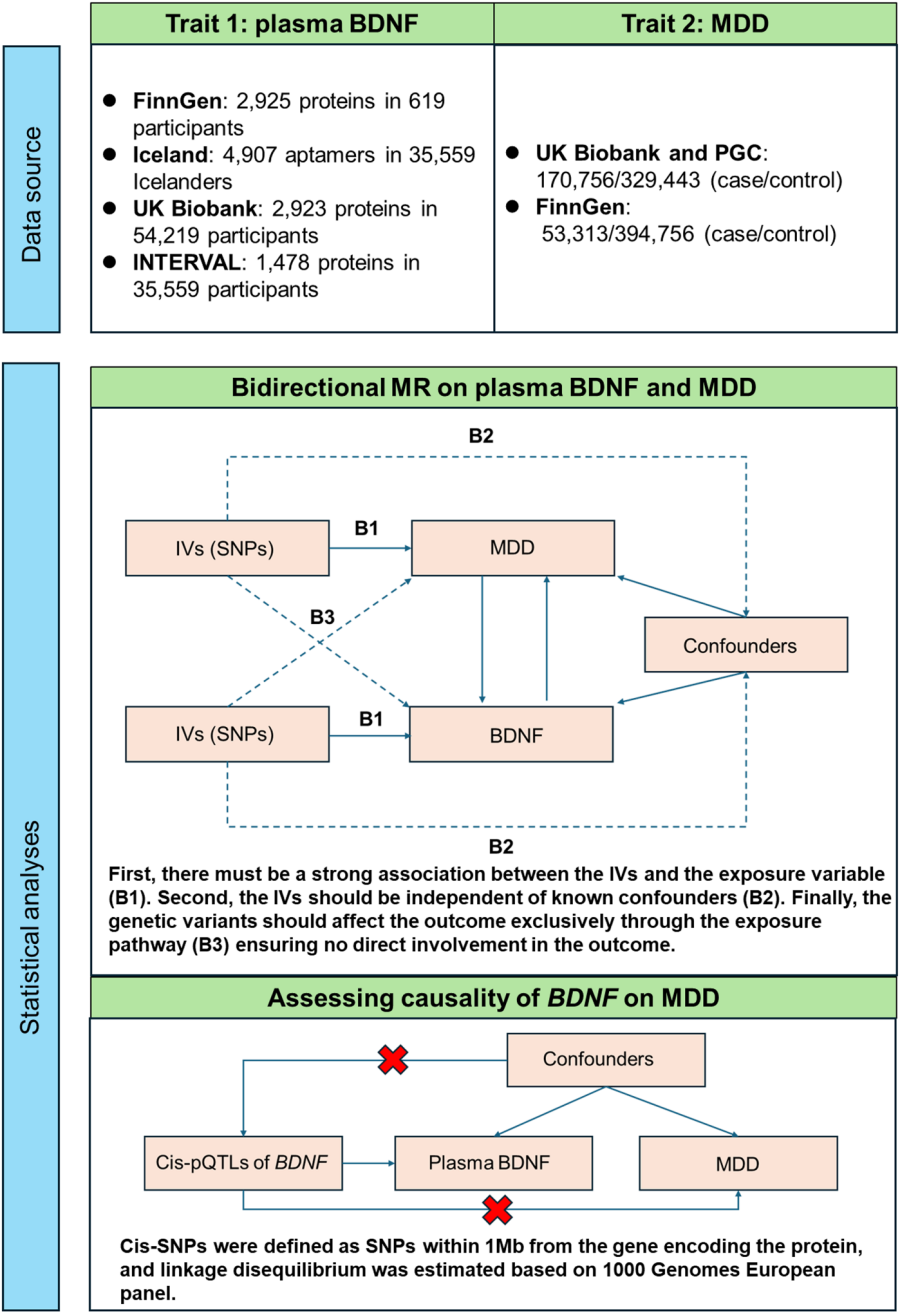
Study design.

We conducted a two‐sample bidirectional MR analysis to assess the relationship between BDNF and the risk of MDD in individuals of European ancestry (Figure 1). To minimize bias from overlapping samples, exposure and outcome data were sourced from multiple databases, with details on data sources and sample sizes provided in Table S1. Ethical approval was obtained for all studies involved, and no additional approval was required for this analysis. The study adhered to the STROBE‐MR guidelines (Skrivankova et al. 2021).

### 2.2 Major Depressive Disorder

The Genome‐Wide Association Study (GWAS) for depression was derived from the Psychiatric Genomics Consortium (PGC). To avoid data redundancy, overlapping information from the UK Biobank and 23andMe was excluded (Howard et al. 2019). An expanded depression phenotype was further investigated by incorporating additional data from the UK Biobank, resulting in a comprehensive dataset of 500,199 individuals, including 170,756 cases and 329,443 controls. In addition, MDD‐related summary data were obtained from the FinnGen Consortium (finn‐b‐F5_DEPRESSIO), which includes 53,313 cases and 394,756 controls.

### 2.3 Plasma Brain‐Derived Neurotrophic Factor

Single nucleotide polymorphisms (SNPs) associated with BDNF levels were selected as instrumental variables from four large‐scale GWASs: the FinnGen study, the UK Biobank Pharma Proteomics Project (UKB‐PPP), the deCODE Health study, and the INTERVAL study. The FinnGen study analyzed proteomic profiling on blood plasma samples from 619 individuals of European descent using the Olink platform, generating data on 2925 proteins (Pietzner et al. 2021). The UKB‐PPP profiled blood plasma samples from 54,219 participants using the Olink platform, gathering data on 1463 proteins. The deCODE Health study utilized the SomaScan platform to assess 4907 aptamers in 35,559 Icelanders (Ferkingstad et al. 2021). The INTERVAL study reported 1927 genetic associations with 1478 proteins, including trans associations for 1104 proteins (Sun et al. 2018).

### 2.4 Genetic Variants Selection Criteria

MR relies on three key assumptions (Burgess et al. 2015). Independent SNPs strongly associated with the exposure were identified using a filtration criterion of *p* < 5 × 10^−8^ within a 10,000 kb window around the lead SNP. This cut‐off value was applied to select instrumental variables (IVs) for MDD (Huang et al. 2023). For BDNF, the significance threshold was adjusted to 5 × 10^−6^ due to the limited availability of instrumental variables. SNPs were selected based on a minor allele frequency (MAF) greater than 0.01. To ensure the validity of the IVs, exposure and outcome effects were harmonized, and SNPs with insufficient F‐statistics were excluded. Steiger filtering was applied to address reverse causation. Each SNP and its proxies (*R* ^2^ > 0.90) were analyzed using LDlink, a web‐based tool that generates haplotype tables and interactive plots based on queries of SNPs in relevant population groups (Machiela and Chanock 2015).

Additionally, the effects of *cis*‐SNPs for *BDNF* and MDD were assessed, with *cis*‐SNPs defined as those located within 1 Mb of the gene encoding the protein. Linkage disequilibrium was estimated using the 1000 Genomes European panel.

### 2.5 Statistical Analysis

The primary MR analysis employed the inverse‐variance weighted (IVW) method to evaluate the causal relationship between BDNF and MDD (Burgess et al. 2013). This method estimates the causal effect from the ratio of SNPs associated with the exposure, assuming random SNP distribution and minimizing reverse causality.

The analysis incorporated IVW, MR‐Egger, weighted median, and weighted mode methods, implemented via the “TwoSampleMR” package in R (version 4.2.3) (Yavorska and Burgess 2017; Pierce and Burgess 2013). A causal link was considered significant with a *p* value below 0.05. MR‐Egger was utilized to address potential confounding and pleiotropy with fewer assumptions, while the weighted median approach provided robust estimates when at least 50% of the genetic variations were valid instruments. The weighting method refined estimates based on weighted analysis. For proteins with only one *cis*‐pQTL, the Wald ratio method was employed to estimate OR and corresponding CI (Pierce and Burgess 2013).

To assess the reliability of the findings, sensitivity analyses were performed using MR‐Egger regression, the weighted median method, and the “leave‐one‐out” approach. The “leave‐one‐out” method sequentially excluded each SNP and recalculated the pooled effect of the remaining SNPs when the IVW method produced significant results (*p* < 0.05) and passed heterogeneity and gene diversity tests.

## 3 Results

### 3.1 Instrumental Variable Selection

A meticulous screening process was conducted to identify SNPs strongly associated with plasma BDNF levels, using criteria such as *p* < 5 × 10^−6^, *F* value > 10, and a MAF > 0.01. This approach ensured the independence of the selected SNPs (*r* ^2^ < 0.001 within a 10,000 kb window), resulting in the exclusion of 9 SNPs from the FinnGen Consortium. The selection was refined through the harmonization of exposure and outcome data, alongside passing the Steiger test, leading to the inclusion of eight SNPs for MR analysis. Additionally, two SNPs associated with confounders were excluded.

For MDD, a similar screening process was implemented, starting with SNPs demonstrating strong associations (*p* < 5 × 10^−8^, *F* value > 10, MAF > 0.01), while maintaining independence (*r* ^2^ < 0.001 within a 10,000 kb window). This resulted in the exclusion of 50 SNPs from the UK Biobank and PGC datasets. Following harmonization and validation through the Steiger test, 11 SNPs were selected for MR analysis, with 1 SNP linked to confounders excluded (Tables S2 and S3).

### 3.2 Causal Effect From BDNF to MDD

**FIGURE 2:**
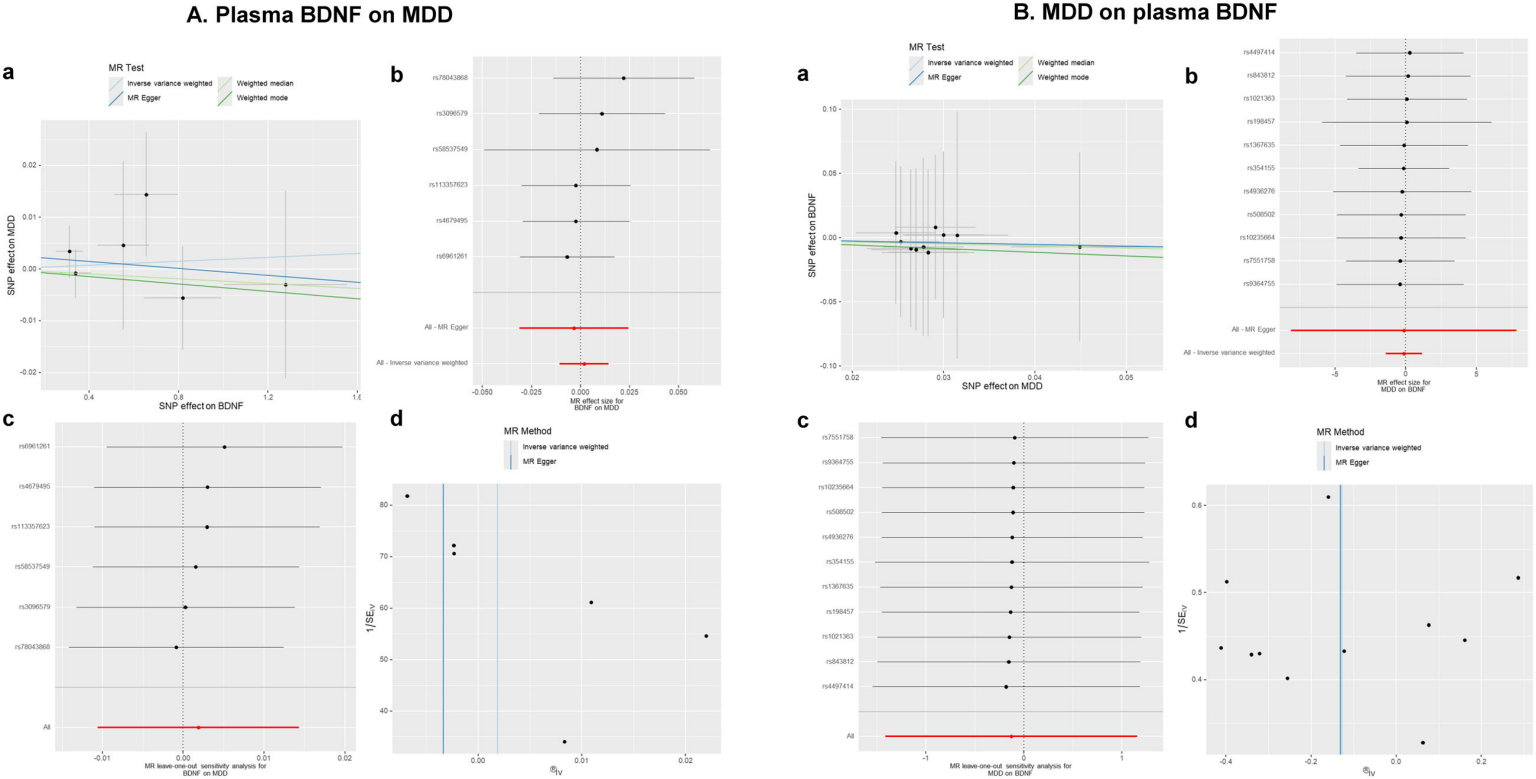
Causal relationship of plasma BDNF and MDD. (a) Scatter plot of exposure effect estimates on outcome. (b) Forest plot summarizing exposure's overall impact on the outcome. (c) Sensitivity analysis via “leave‐one‐out” plots. (d) Funnel plot for bias assessment of the estimates.

**FIGURE 3:**
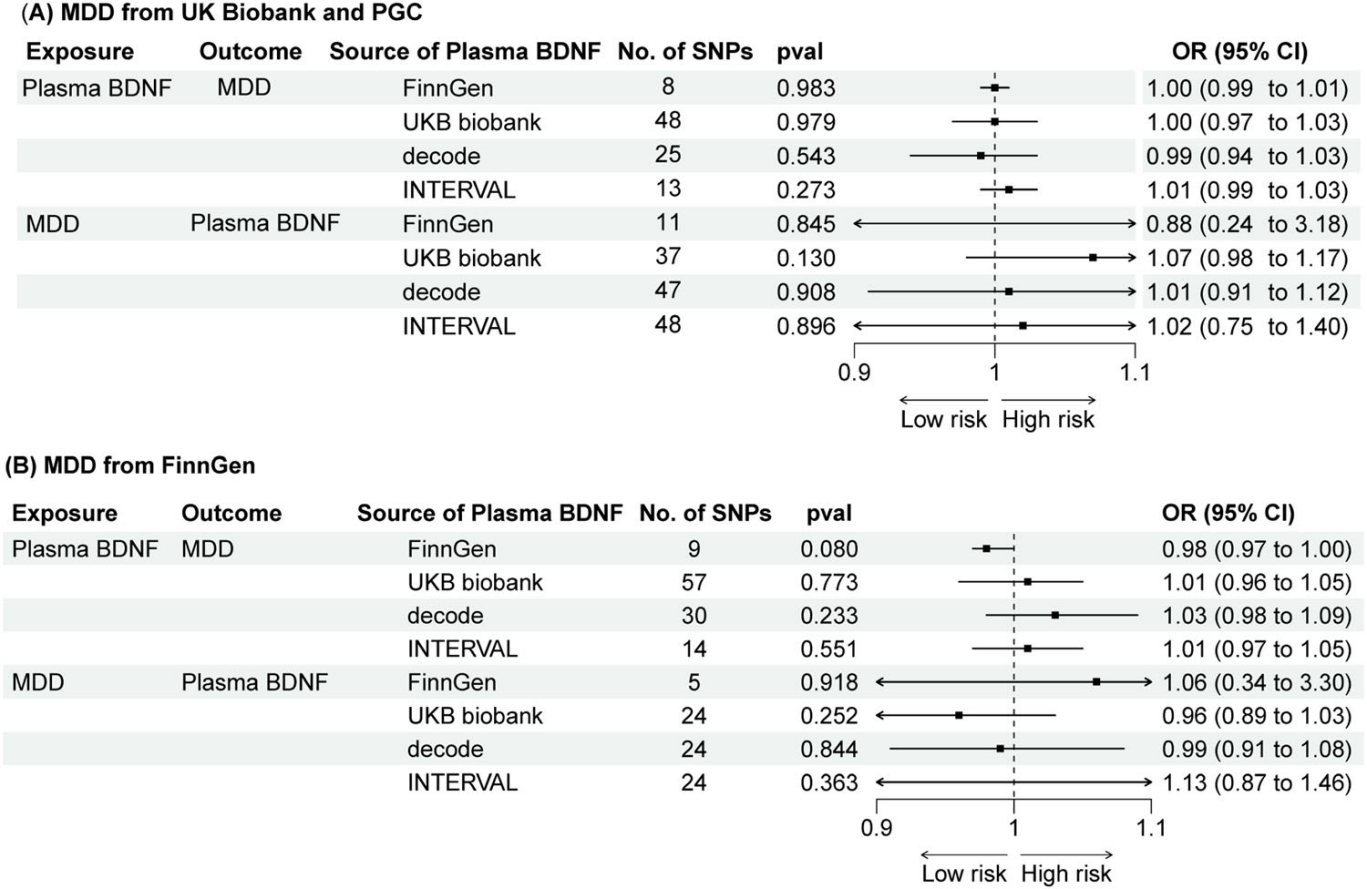
Causal relationship of MDD and plasma BDNF in subsequent validation analysis.

The IVW method revealed no significant association between genetic predisposition to plasma BDNF levels and MDD in the MR analysis (OR = 1.00; 95% CI = 0.99 to 1.01; *p* = 0.769). The MR‐Egger intercept indicated no evidence of directional pleiotropy (*p* = 0.701), and Cochran's Q test showed no evidence of heterogeneity (*p* = 0.814) (Table 1, Figure 2). These findings remained consistent during the second phase of the analysis (see Figure 3).

**TABLE 1:** MR results for the relationship between plasma BDNF on MDD.

Exposures	Outcomes	No. of SNPs	Method	OR (95%CI)	*p*	Heterogeneity test	Pleiotropy test
BDNF^a^	MDD^b^	6^c^	IVW	1.00 (0.99–1.01)	0.769	0.814	0.701
			MR Egger	1.00 (0.97–1.02)	0.825		
			Weighted median	1.00 (0.98–1.01)	0.761		
			Weighted mode	1.00 (0.98–1.02)	0.732		
			MR presso	1.00 (0.99–1.01)	0.680		
MDD^b^	BDNF^a^	10^d^	IVW	0.91 (0.23–3.56)	0.893	0.999	0.989
			MR Egger	0.86 (0.00–2507)	0.971		
			Weighted median	0.87 (0.17–4.32)	0.861		
			Weighted mode	0.80 (0.07–9.02)	0.859		
			MR presso	0.91 (0.79–1.06)	0.248		
*BDNF* ^e^	MDD^b^	2	IVW	0.99 (0.89–1.09)	0.783		

^a^ Data form the FinnGen Consortium.

^b^ Data form PGC and UK Biobank.

^c^ Two SNPs linked to SCZ and AD were omitted.

^d^ One SNP linked to BMI was omitted.

^e^ Using *cis*‐SNPs of *BDNF* form the FinnGen Consortium.

**FIGURE 4:**
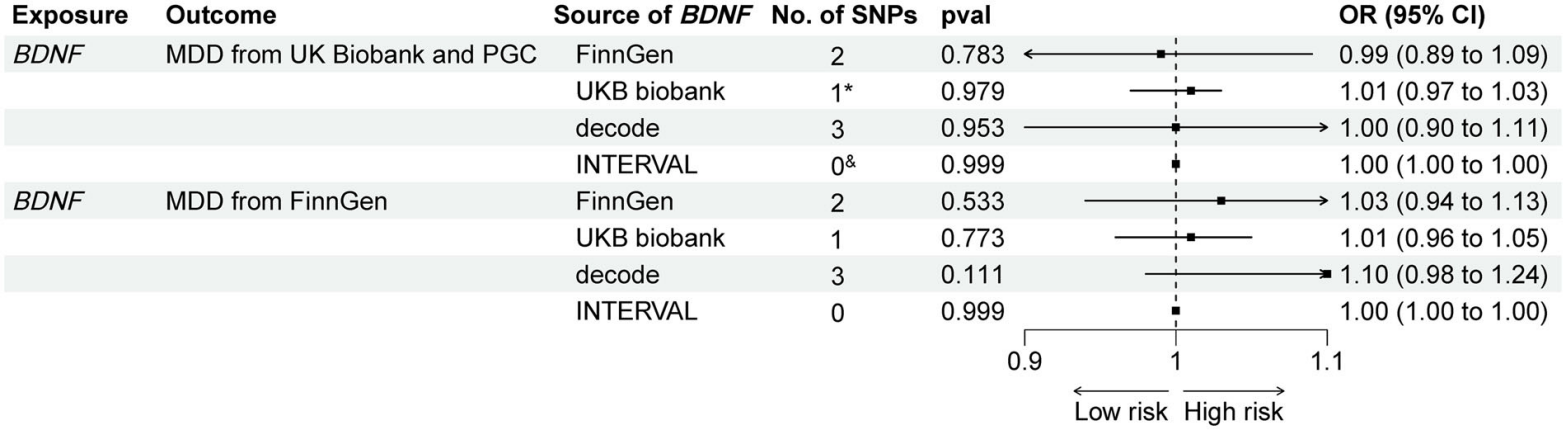
Causal effect of *BDNF* on MDD in subsequent validation analysis. *cis*‐pQTLs are located near the genes that encode proteins. *For proteins with only one *cis*‐pQTL, the Wald ratio method was employed. ^&^ No *cis*‐pQTLs.

Using *cis*‐SNPs as instrumental variables confirmed no causal relationship between *BDNF* and MDD, with consistent results in the second phase of analysis (Figure 4). The findings remained consistent even when SNPs associated with potential confounding factors were included (Table S4 and Figure S1 *)*.

### 3.3 Causal Effect From MDD to BDNF

The IVW method indicated that genetic predisposition to MDD did not show a significant association with plasma BDNF levels in the MR analysis (OR = 0.91; 95% CI = 0.23 to 3.56; *p* = 0.893) (Table 1). These findings were consistent in the second phase of the analysis (Figure 3). The results remained consistent even when SNPs associated with potential confounding factors were not excluded (Table S5 and Figure S2).

## 4 Discussion

A bidirectional MR analysis using publicly available GWAS data was conducted to investigate the causal relationship between BDNF and MDD. While previous research suggested an association between plasma BDNF levels and MDD, this study found no evidence of a causal effect of plasma BDNF on MDD risk. Moreover, the MR analysis showed no indication that genetic susceptibility to MDD influences BDNF levels.

MDD is a significant global health challenge affecting hundreds of millions of people (Ferrari et al. 2013). BDNF plays a crucial role in the survival, growth, and maintenance of neurons that regulate emotional and cognitive functions (Phillips 2017). Accumulating evidence indicates that neuroplastic mechanisms mediated by BDNF are disrupted in both MDD and stress‐induced animal models (Duman and Monteggia 2006; Page et al. 2024). Clinical and preclinical studies suggest that depressive pathology related to stress impacts BDNF levels and function, leading to disruptions in neuroplasticity at both regional and circuit levels in individuals with MDD (Zhang et al. 2016). Conversely, treatments that alleviate depressive symptoms—such as antidepressants and physical activity—have been shown to enhance BDNF levels in key brain regions. These treatments not only support neuronal health but also promote the recovery of MDD‐related circuits and enhance the efficacy of pharmacotherapy.

Although previous observational studies have established correlations between plasma BDNF levels and MDD, the causal role of genetically predicted plasma BDNF in the development of MDD remains unverified. MR studies, which use instrumental variables to infer causality between intermediate phenotypes and disease states, have become increasingly prevalent in MDD research. MR mimics a randomized controlled trial by leveraging Mendel's second law, which states that alleles for different genes are independently inherited during gametogenesis. Thus, the efficacy of MR analyses relies on the assumption that the inheritance of one trait is independent of the inheritance of others (Davey Smith and Ebrahim 2005).

Several mechanisms may underline the relationship between BDNF and MDD (Aldoghachi et al. 2019; Hu et al. 2021; Kishi et al. 2017). First, while this analysis did not find a significant link between genetic liability for MDD and plasma BDNF levels, this does not rule out the possibility that plasma BDNF may still be a valuable predictor of MDD risk. Second, it is crucial to consider that plasma BDNF could play an important role in predicting MDD risk, and the current study may not fully capture all relevant factors. The biological role of BDNF is inherently complex and may be impacted by various factors, including epigenetic modifications (X. M. Zhang, J. Huang, et al. 2024; Xing et al. 2021). Future research should investigate this relationship from multiple perspectives, with a particular emphasis on the functional roles of BDNF in plasma and their clinical implications for BDNF‐targeted therapeutic strategies in MDD (Dwivedi et al. 2003; Siuciak et al. 1997; Shirayama et al. 2002; Karege et al. 2005). Finally, postmortem studies have reported lower BDNF protein levels in the hippocampus and prefrontal cortex of individuals with psychiatric disorders who have died by suicide, compared to non‐psychiatric controls. These findings suggest that BDNF level alterations in brain are more closely associated with MDD than plasma levels. Continued research should focus on BDNF's role in the central nervous system to deepen understanding of its involvement in MDD.

This MR study offers several significant strengths. First, four complementary MR methods were employed to mitigate the risk of reverse causation bias, ensuring robust and reliable results. Second, a diverse array of SNPs was used as instrumental variables to explore the relationship between plasma BDNF and MDD risk, enhancing the sensitivity of the analysis and providing a comprehensive genetic perspective on plasma BDNF. Third, the instrumental variables were carefully selected independent SNPs, reducing potential interference from linkage disequilibrium and improving the precision of our estimates. Overall, the findings suggest that genetically predicted plasma BDNF does not have a causal effect on MDD risk.

## 5 Limitations

This study has several limitations. First, it focuses exclusively on individuals of European ancestry, which may limit the applicability of the findings to non‐European populations. To improve the generalizability across diverse genetic backgrounds, further MR studies involving a wider variety of ethnic groups are recommended. Second, the inherent complexity of biological systems may affect the precision of our findings. The analysis specifically examined plasma BDNF levels, excluding potential associations between BDNF levels in cerebrospinal fluid or brain tissue and MDD. Therefore, additional research is needed to explore these relationships within different biological contexts.

## 6 Conclusion

In this study, MR analysis was employed to investigate potential causal links between plasma BDNF and MDD. Despite thorough efforts to elucidate causal mechanisms connecting these traits, the analysis concluded that plasma BDNF does not have a causal association with MDD.

## Author Contributions

**Ming Chen**: conceptualization, writing–review and editing, writing–original draft. **Hao‐Zhang Huang**: conceptualization, methodology, software, data curation, investigation, validation, formal analysis, supervision, visualization, writing–review and editing, writing–original draft, funding acquisition. **Yi‐Hui Liu**: conceptualization, methodology, validation, software. **Qiang Li**: writing–review and editing. **Lin‐Yan Fu**: writing–review and editing, conceptualization, methodology, formal analysis, project administration, resources, supervision. **Cai‐Lan Hou**: conceptualization, methodology, writing–review and editing, project administration, resources, supervision, formal analysis.

## Ethics Statement

The authors have nothing to report.

## Consent

The authors have nothing to report.

## Conflicts of Interest

The authors declare no conflicts of interest.

### Peer Review

The peer review history for this article is available at https://publons.com/publon/10.1002/brb3.70425

## Supporting information



Supplement Table 1 Details of the GWASs included in the Mendelian randomization.
Supplement Table 2 Nine index SNPs represented genetically predicted BDNF from FinnGen.
Supplementary Table 3. 50 index SNPs represented genetically predicted MDD from UK Biobank and PGC.
Supplement Table 4 MR Results for the Relationship Between Plasma BDNF on MDD without Omitting SNPs about Confounders.
Supplement Table 5 MR Results for the Relationship Between MDD on plasma BDNF without Omitting SNPs about Confounders.
Supplement Figure 1 Causal effects of BDNF on MDD without omitting SNPs about confounders.
Supplement Figure 2 Causal effects of MDD on plasma BDNF without omitting SNPs about confounders.

Supporting Information

## Data Availability

The data that support the findings of this study are available in Psychiatric Genomics Consortium at https://pgc.unc.edu/. These data were derived from the following resources available in the public domain: PGC “Psychiatric Genomics Consortium,” https://pgc.unc.edu/for‐researchers/download‐results/.
