# Supplementary material for: Assessing Causality Between Plasma Brain‐Derived Neurotrophic Factor With Major Depression Disorder: A Bidirectional Mendelian Randomization Study
Source: Brain Behav. 2025 Mar 18;15(3):e70425. doi: 10.1002/brb3.70425 (PMC11919739; doi:10.1002/brb3.70425)

**Supplement Table 1 Details of the GWASs included in the Mendelian randomization.**

| Phenotype | Consortium | Participants | platform | Ancestry | Year | PubMed identifier/ Web source |
| --- | --- | --- | --- | --- | --- | --- |
| Major depressive disorder | UK Biobank and PGC | 170,756/329,443 | - | European | 2019 | 30718901 |
| Major depressive disorder | FinnGen | 53,313/ 394,756 | - | European | 2024 | https://r10.finngen.fi/ |
| BDNF | FinnGen |  | Olink | European | 2021 | 34648354 |
| BDNF | deCODE | 619 | Somascan | European | 2021 | 34857953 |
| BDNF | UK Biobank | 54,306 | Olink | European | 2023 | 37794186 |
| BDNF | INTERVAL | 35,559 | Somascan | European | 2018 | 29875488 |

**Supplement Table 2 Nine index SNPs represented genetically predicted BDNF from FinnGen.**

| SNP | effect allele | other allele | beta | se | pval | eaf |
| --- | --- | --- | --- | --- | --- | --- |
| rs7225724 | G | A | 0.361294 | 0.0749907 | 1.83E-06 | 0.842488 |
| rs78043868 | G | A | -0.655624 | 0.142047 | 4.77E-06 | 0.0403877 |
| rs113357623 | T | C | -1.2779 | 0.27571 | 4.36E-06 | 0.0105008 |
| rs4679495 | A | G | 0.339266 | 0.0702658 | 1.74E-06 | 0.804523 |
| rs73793590 | T | C | 1.63747 | 0.349908 | 3.53E-06 | 0.00646204 |
| rs3096579 | T | C | -0.31198 | 0.0616931 | 5.62E-07 | 0.258481 |
| rs6961261 | G | C | 0.817962 | 0.17282 | 2.75E-06 | 0.0258481 |
| rs58537549 | G | A | 0.55225 | 0.114635 | 1.83E-06 | 0.0621971 |
| rs561618 | G | A | 0.280451 | 0.0599723 | 3.59E-06 | 0.344103 |

**Supplementary Table 3. 50 index SNPs represented genetically predicted MDD from UK Biobank and PGC.**

| SNP | effect allele | other allele | beta | se | pval | eaf |
| --- | --- | --- | --- | --- | --- | --- |
| rs7551758 | G | T | 0.0283 | 0.0043 | 5.11E-11 | 0.5329 |
| rs2568958 | A | G | 0.0382 | 0.0044 | 2.90E-18 | 0.6042 |
| rs10913112 | T | C | -0.0262 | 0.0045 | 4.53E-09 | 0.378 |
| rs17641524 | T | C | -0.03 | 0.0053 | 1.50E-08 | 0.2101 |
| rs354155 | C | G | -0.0449 | 0.0075 | 1.75E-09 | 0.0923 |
| rs7538938 | C | T | 0.0251 | 0.0043 | 7.29E-09 | 0.5599 |
| rs4141983 | C | T | -0.0264 | 0.0046 | 9.69E-09 | 0.326 |
| rs2111592 | A | G | 0.0263 | 0.0046 | 1.35E-08 | 0.3141 |
| rs72948506 | A | G | 0.0265 | 0.0047 | 1.71E-08 | 0.2975 |
| rs35469634 | G | A | -0.0241 | 0.0044 | 3.28E-08 | 0.5774 |
| rs843812 | A | G | 0.0248 | 0.0044 | 1.41E-08 | 0.4117 |
| rs9831648 | T | G | -0.0292 | 0.0052 | 1.59E-08 | 0.7739 |
| rs66511648 | C | T | 0.0297 | 0.0048 | 6.03E-10 | 0.284 |
| rs76954012 | A | T | 0.0412 | 0.0074 | 2.41E-08 | 0.0931 |
| rs30266 | A | G | 0.0366 | 0.0046 | 1.43E-15 | 0.3271 |
| rs247910 | G | A | 0.0237 | 0.0043 | 4.71E-08 | 0.457 |
| rs7725715 | A | G | 0.029 | 0.0043 | 1.61E-11 | 0.5343 |
| rs150186873 | C | A | 0.0704 | 0.012 | 4.51E-09 | 0.0327 |
| rs2232423 | G | A | -0.062 | 0.007 | 1.14E-18 | 0.1056 |
| rs9364755 | G | A | 0.0283 | 0.0051 | 3.49E-08 | 0.2262 |
| rs2214123 | G | A | -0.0261 | 0.0045 | 8.56E-09 | 0.6466 |
| rs2876520 | G | C | 0.026 | 0.0043 | 2.24E-09 | 0.4688 |
| rs2522831 | C | T | 0.024 | 0.0043 | 2.11E-08 | 0.4739 |
| rs4730387 | A | T | 0.0238 | 0.0043 | 4.12E-08 | 0.4659 |
| rs150346963 | T | C | 0.0283 | 0.0044 | 1.16E-10 | 0.4118 |
| rs3807865 | A | G | 0.031 | 0.0044 | 1.09E-12 | 0.4105 |
| rs10235664 | C | T | -0.027 | 0.0049 | 4.68E-08 | 0.2529 |
| rs59082935 | T | C | 0.0363 | 0.0066 | 3.07E-08 | 0.1342 |
| rs62535714 | A | G | 0.0339 | 0.0058 | 4.69E-09 | 0.1639 |
| rs1931388 | G | A | -0.0295 | 0.0044 | 1.68E-11 | 0.4042 |
| rs59283172 | A | G | -0.039 | 0.007 | 2.41E-08 | 0.1081 |
| rs2418449 | C | T | -0.0281 | 0.0048 | 4.25E-09 | 0.281 |
| rs1021363 | G | A | -0.03 | 0.0045 | 2.29E-11 | 0.6434 |
| rs198457 | T | C | -0.0315 | 0.0056 | 1.90E-08 | 0.1886 |
| rs4497414 | C | T | 0.0291 | 0.0044 | 2.93E-11 | 0.44 |
| rs4936276 | C | G | 0.0278 | 0.0044 | 3.57E-10 | 0.622 |
| rs61914045 | A | G | 0.0309 | 0.0054 | 7.96E-09 | 0.2034 |
| rs9529218 | T | C | -0.034 | 0.0054 | 2.23E-10 | 0.2031 |
| rs9536381 | T | C | 0.0255 | 0.0046 | 2.62E-08 | 0.3259 |
| rs508502 | T | C | -0.0264 | 0.0048 | 3.56E-08 | 0.2992 |
| rs1950829 | G | A | -0.0297 | 0.0043 | 4.74E-12 | 0.5173 |
| rs754287 | A | T | -0.0289 | 0.0045 | 1.31E-10 | 0.3664 |
| rs7152906 | C | T | 0.0258 | 0.0043 | 1.87E-09 | 0.5196 |
| rs28541419 | G | C | -0.0292 | 0.0052 | 1.76E-08 | 0.2308 |
| rs12919291 | C | G | 0.0327 | 0.0055 | 3.09E-09 | 0.1884 |
| rs4799949 | T | C | -0.0292 | 0.0046 | 1.40E-10 | 0.6684 |
| rs12967143 | C | G | -0.0345 | 0.0047 | 2.53E-13 | 0.7012 |
| rs7241572 | A | G | 0.0323 | 0.0054 | 2.43E-09 | 0.2047 |
| rs1367635 | C | T | 0.0253 | 0.0043 | 4.35E-09 | 0.5148 |
| rs13037326 | T | C | 0.031 | 0.0049 | 2.40E-10 | 0.2597 |

**Supplement Table 4 MR Results for the Relationship Between Plasma BDNF on MDD without Omitting SNPs about Confounders.**

| Exposures | Outcomes | No. of SNPs | Method | OR (95%CI) | p | Heterogeneity test | Pleiotropy test |
| --- | --- | --- | --- | --- | --- | --- | --- |
| BDNF ^a^ | MDD^b^ | 8 | IVW | 1.00 (0.99-1.01) | 0.983 | 0.901 | 0.926 |
|  |  |  | MR Egger | 1.00 (0.98-1.03) | 0.940 |  |  |
|  |  |  | Weighted median | 1.00 (0.98-1.01) | 0.749 |  |  |
|  |  |  | Weighted mode | 1.00 (0.98-1.01) | 0.644 |  |  |
|  |  |  | MR presso | 1.00 (0.99-1.01) | 0.974 |  |  |

^a^ Data form The FinnGen Consortium.

^b^ Data form PGC and UK Biobank.

**Supplement Table 5 MR Results for the Relationship Between MDD on plasma BDNF without Omitting SNPs about Confounders.**

| Exposures | Outcomes | No. of SNPs | Method | OR (95%CI) | p | Heterogeneity test | Pleiotropy test |
| --- | --- | --- | --- | --- | --- | --- | --- |
| MDD | BDNF | 11 | IVW | 0.88 (0.24-3.18) | 0.845 | 0.999 | 0.999 |
|  |  |  | MR Egger | 0.88 (0.00-2545) | 0.975 |  |  |
|  |  |  | Weighted median | 0.85 (0.17-4.18) | 0.842 |  |  |
|  |  |  | Weighted mode | 0.75 (0.09-6.60) | 0.804 |  |  |
|  |  |  | MR presso | 0.88 (0.76-1.02) | 0.115 |  |  |

**Supplement Figure 1 Causal effects of BDNF on MDD without omitting SNPs about confounders.**


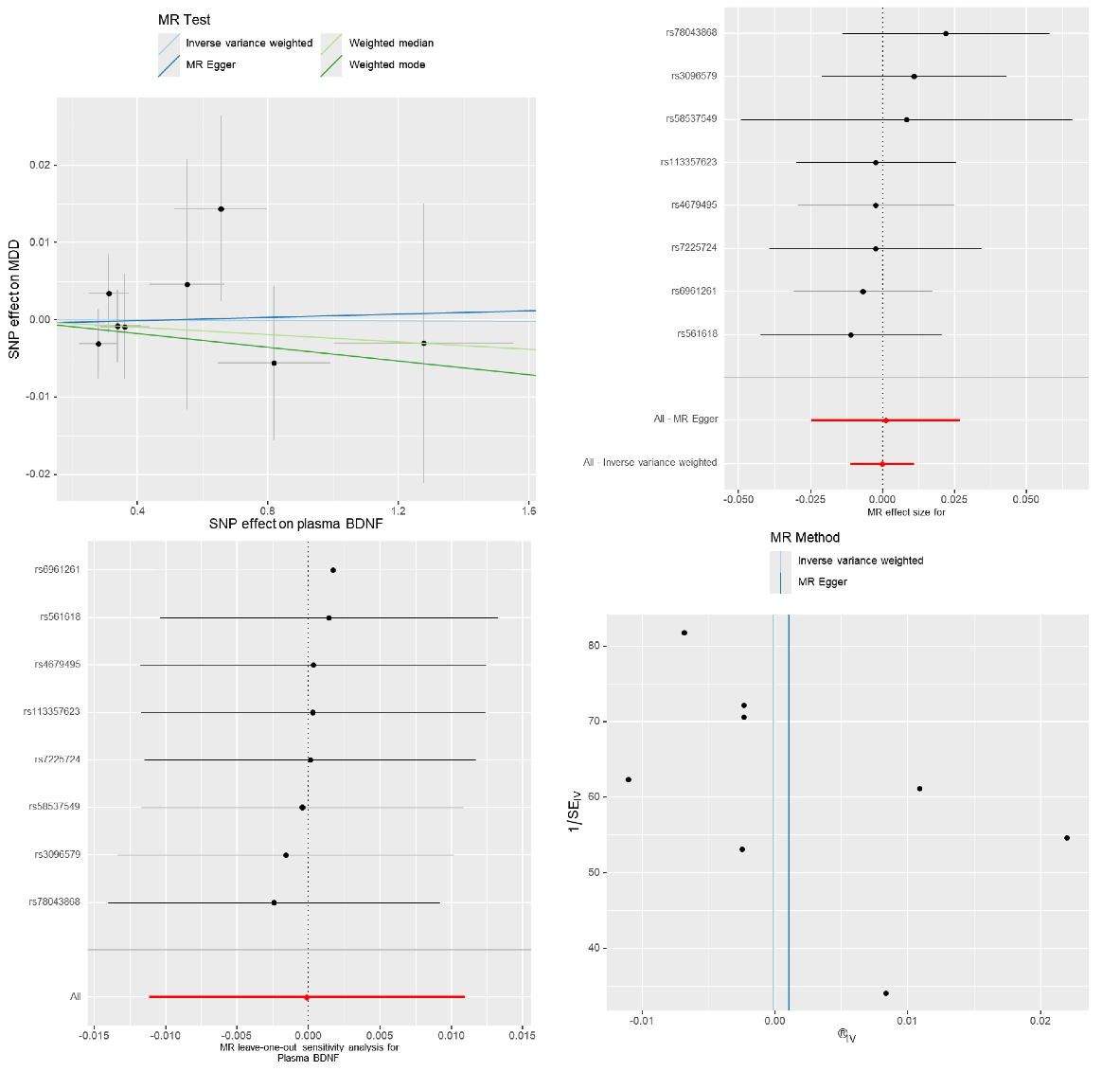


**Supplement Figure 2 Causal effects of MDD on plasma BDNF without omitting SNPs about confounders.**


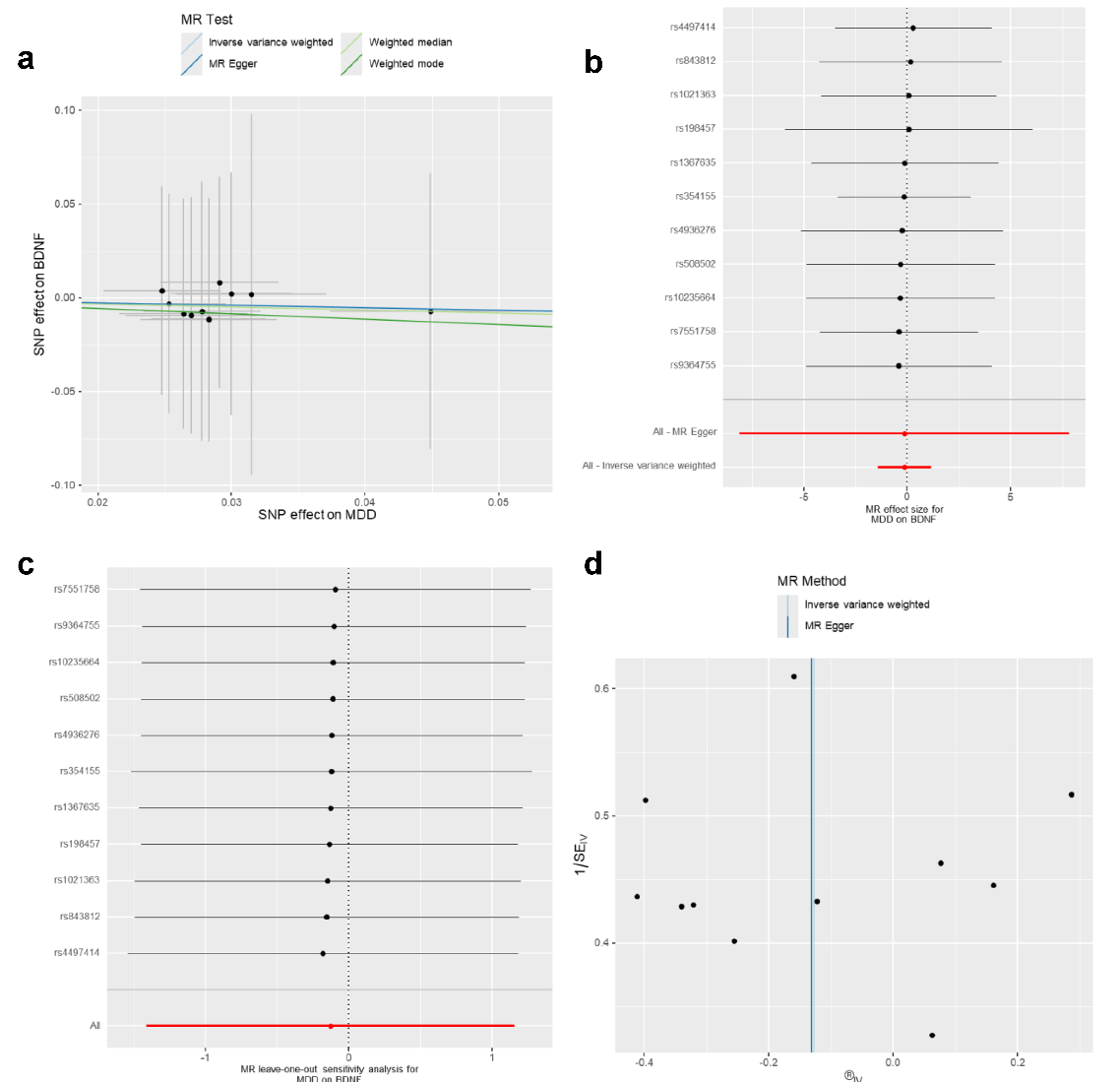

Supplement: Supplementary file 1 — Supplement Table 1 Details of the GWASs included in the Mendelian randomization. Supplement Table 2 Nine index SNPs represented genetically predicted BDNF from FinnGen. Supplementary Table 3. 50 index SNPs represented genetically predicted MDD from UK Biobank and PGC. Supplement Table 4 MR Results for the Relationship Between Plasma BDNF on MDD without Omitting SNPs about Confounders. Supplement Table 5 MR Results for the Relationship Between MDD on plasma BDNF without Omitting SNPs about Confounders. Supplement Figure 1 Causal effects of BDNF on MDD without omitting SNPs about confounders. Supplement Figure 2 Causal effects of MDD on plasma BDNF without omitting SNPs about confounders. [file BRB3-15-e70425-s002.docx]
