# Supplementary material for: Assessing Causality Between Plasma Brain‐Derived Neurotrophic Factor With Major Depression Disorder: A Bidirectional Mendelian Randomization Study
Source: Brain Behav. 2025 Mar 18;15(3):e70425. doi: 10.1002/brb3.70425 (PMC11919739; doi:10.1002/brb3.70425)
Supplement: Supplementary file 2 — Supporting Information [file BRB3-15-e70425-s001.docx]

**STROBE-MR checklist of recommended items to address in reports of Mendelian randomization studies**^1^ ^2^

| **Item No.** | **Section** | **Checklist item** | **Page No.** | **Relevant text from manuscript** |
| --- | --- | --- | --- | --- |
| 1 | **TITLE and ABSTRACT** | Indicate Mendelian randomization (MR) as the study’s design in the title and/or the abstract if that is a main purpose of the study | 1-2 | Title: Assessing causality between plasma brain-derived neurotrophic factor with major depression disorder: A bidirectional Mendelian randomization study  ABSTRACT:Purpose: This study employed a two-sample Mendelian randomization (MR) approach to investigate the bidirectional relationship between brain-derived neurotrophic factor (BDNF) and major depressive disorder (MDD), addressing gaps left by previous observational studies. |
|  | **INTRODUCTION** |  |  |  |
| 2 | **Background** | Explain the scientific background and rationale for the reported study. What is the exposure? Is a potential causal relationship between exposure and outcome plausible? Justify why MR is a helpful method to address the study question | 3 | Accumulating evidence associates reduced central and plasma BDNF levels with various neuropsychiatric disorders, suggesting its potential as a biomarker for central pathologies [3, 4]. Previous studies have shown that decreased plasma levels of BDNF are strong indicators for predicting the occurrence of MDD [5, 6]. Previous studies have shown that decreased plasma levels of BDNF are strong indicators for predicting the occurrence of MDD [7]. However, despite evidence pointing to the relationship between plasma BDNF and MDD, several factors, such as heterogeneous results, small sample sizes, publication bias, and differences in BDNF measurements (serum or plasma), pose challenges in interpreting this relationship. |
| 3 | **Objectives** | State specific objectives clearly, including pre-specified causal hypotheses (if any). State that MR is a method that, under specific assumptions, intends to estimate causal effects | 3 | Thus, we conducted a bidirectional MR analysis to determine if genetic variability in MDD patients is causally linked to plasma BDNF levels. Understanding this bidirectional relationship is vital for comprehending the complex interactions underlying these conditions. |
|  | **METHODS** |  |  |  |
| 4 | **Study design and data sources** | Present key elements of the study design early in the article. Consider including a table listing sources of data for all phases of the study. For each data source contributing to the analysis, describe the following: | 3 | Supplement Table 1 provides details on the data sources and sample sizes. |
|  | a) | Setting: Describe the study design and the underlying population, if possible. Describe the setting, locations, and relevant dates, including periods of recruitment, exposure, follow-up, and data collection, when available. | 3 | We conducted a two-sample bidirectional Mendelian randomization (MR) analysis to assess the relationship between BDNF and MDD risk in individuals of European ancestry |
|  | b) | Participants: Give the eligibility criteria, and the sources and methods of selection of participants. Report the sample size, and whether any power or sample size calculations were carried out prior to the main analysis | 3 | Supplement Table 1 provides details on the data sources and sample sizes. |
|  | c) | Describe measurement, quality control and selection of genetic variants | 3 | Supplement Table 1 provides details on the data sources and sample sizes. |
|  | d) | For each exposure, outcome, and other relevant variables, describe methods of assessment and diagnostic criteria for diseases | 3 | Supplement Table 1 provides details on the data sources and sample sizes. |
|  | e) | Provide details of ethics committee approval and participant informed consent, if relevant | 4 | Ethical approval was granted for all studies, and no additional approval was necessary for this analysis. |
| 5 | **Assumptions** | Explicitly state the three core IV assumptions for the main analysis (relevance, independence and exclusion restriction) as well assumptions for any additional or sensitivity analysis | 4 | Mendelian randomization relies on three core assumptions [23]: (1) the selected instrumental variables (IVs) (single nucleotide polymorphisms, SNPs) must be strongly associated with the exposure; (2) the genetic variants should not be associated with confounders that influence both the exposure and outcome; and (3) the genetic variants should only affect the outcome through the exposure and not via other pathways |
| 6 | **Statistical methods: main analysis** | Describe statistical methods and statistics used |  |  |
|  | a) | Describe how quantitative variables were handled in the analyses (i.e., scale, units, model) | NA |  |
|  | b) | Describe how genetic variants were handled in the analyses and, if applicable, how their weights were selected | 5 | We selected independent SNPs strongly associated with the exposure by applying a filtration criterion of P < 5 × 10-8 within a 10,000 kb window around the lead SNP. This cut-off value was used to identify instrumental variables (IVs) for MDD [19]. For BDNF, we adjusted the significance threshold to 5 × 10-6 due to a smaller number of available instrumental variables. SNPs were selected based on a minor allele frequency (MAF) greater than 0.01. We ensured the validity of the instrumental variables by harmonizing exposure and outcome effects and excluding SNPs with insufficient F-statistics. To address potential reverse causation, we applied Steiger filtering. Each SNP and its proxies (R² > 0.90) were analyzed using LDlink, a web-based tool that generates haplotype tables and interactive plots by querying SNPs in relevant population groups [20]. We also evaluated the effects of Cis-SNPs for BDNF and MDD, where Cis-SNPs were defined as those located within 1 Mb of the gene encoding the protein. Linkage disequilibrium was estimated using the 1000 Genomes European panel. |
|  | c) | Describe the MR estimator (e.g. two-stage least squares, Wald ratio) and related statistics. Detail the included covariates and, in case of two-sample MR, whether the same covariate set was used for adjustment in the two samples | 5 | We conducted the primary Mendelian randomization (MR) analysis using the inverse-variance weighted (IVW) method to evaluate the causal relationship between BDNF and MDD [21]. This method estimates the causal effect from the ratio of SNPs associated with the exposure, assuming random SNP distribution and minimizing reverse causality. |
|  | d) | Explain how missing data were addressed | NA |  |
|  | e) | If applicable, indicate how multiple testing was addressed | NA |  |
| 7 | **Assessment of assumptions** | Describe any methods or prior knowledge used to assess the assumptions or justify their validity |  |  |
| 8 | **Sensitivity analyses and additional analyses** | Describe any sensitivity analyses or additional analyses performed (e.g. comparison of effect estimates from different approaches, independent replication, bias analytic techniques, validation of instruments, simulations) | 5 | Our analysis included IVW, MR-Egger, weighted median, and weighted mode methods, implemented via the "TwoSampleMR" package in R (version 4.0.3) [22, 23]. A causal link was considered significant with a p-value below 0.05. MR-Egger was used to address potential confounding and pleiotropy with fewer assumptions, while the weighted median approach ensured robust estimates when at least 50% of genetic variations were valid instruments. The weighting method refined estimates based on weighted analysis. |
| 9 | **Software and pre-registration** |  |  |  |
|  | a) | Name statistical software and package(s), including version and settings used | 5 | Our analysis included IVW, MR-Egger, weighted median, and weighted mode methods, implemented via the "TwoSampleMR" package in R (version 4.0.3) [22, 23]. |
|  | b) | State whether the study protocol and details were pre-registered (as well as when and where) | NA |  |
|  | **RESULTS** |  |  |  |
| 10 | **Descriptive data** |  |  |  |
|  | a) | Report the numbers of individuals at each stage of included studies and reasons for exclusion. Consider use of a flow diagram | 6 | A meticulous screening process was conducted to select SNPs strongly associated with plasma BDNF levels, using criteria such as P < 5 × 10⁶, F-value > 10, and MAF > 0.01. This process ensured the independence of the SNPs (r² < 0.001, kb = 10000) and resulted in the exclusion of 9 SNPs from the FinnGen Consortium. The harmonization of exposure and outcome data, alongside passing the Steiger test, refined the selection further, leading to 8 SNPs being chosen for the Mendelian randomization (MR) analysis. Additionally, 2 SNPs associated with confounders were excluded.  For MDD, a similar process was followed, starting with SNPs exhibiting strong associations (P < 5 × 10⁸, F-value > 10, MAF > 0.01) and maintaining independence (r² < 0.001, kb = 10000). This led to the exclusion of 50 SNPs from the UK Biobank and PGC datasets. After harmonization and Steiger test validation, 11 SNPs were selected for the MR analysis, with 1 SNP associated with confounders being excluded (Supplementary Tables 2–3). |
|  | b) | Report summary statistics for phenotypic exposure(s), outcome(s), and other relevant variables (e.g. means, SDs, proportions) | 3 | Supplement Table 1 provides details on the data sources and sample sizes. |
|  | c) | If the data sources include meta-analyses of previous studies, provide the assessments of heterogeneity across these studies | NA |  |
|  | d) | For two-sample MR:  i.  Provide justification of the similarity of the genetic variant-exposure associations between the exposure and outcome samples  ii.  Provide information on the number of individuals who overlap between the exposure and outcome studies | 6 | The IVW method revealed no significant association between genetic predisposition to plasma BDNF levels and MDD in the MR analysis (OR = 1.00; 95% CI = 0.99 to 1.01; P = 0.769). The MR-Egger intercept also indicated no evidence of directional pleiotropy (P = 0.701), and Cochran's Q test showed no evidence of heterogeneity (P = 0.814) (Table 1, Fig. 2). The use of cis-SNPs as instrumental variables confirmed that there is no causal relationship between BDNF and MDD, with consistent results obtained in the second phase of analysis (Figs. 3 and 4). The results remained consistent even when SNPs associated with potential confounding factors were not excluded (Supplement Table 4 and Supplement Figure 1). |
| 11 | **Main results** |  |  |  |
|  | a) | Report the associations between genetic variant and exposure, and between genetic variant and outcome, preferably on an interpretable scale | 6 | For MDD, a similar process was followed, starting with SNPs exhibiting strong associations (P < 5 × 10⁸, F-value > 10, MAF > 0.01) and maintaining independence (r² < 0.001, kb = 10000). This led to the exclusion of 50 SNPs from the UK Biobank and PGC datasets. After harmonization and Steiger test validation, 11 SNPs were selected for the MR analysis, with 1 SNP associated with confounders being excluded (Supplementary Tables 2–3). |
|  | b) | Report MR estimates of the relationship between exposure and outcome, and the measures of uncertainty from the MR analysis, on an interpretable scale, such as odds ratio or relative risk per SD difference | 6 | The IVW method revealed no significant association between genetic predisposition to plasma BDNF levels and MDD in the MR analysis (OR = 1.00; 95% CI = 0.99 to 1.01; P = 0.769). The MR-Egger intercept also indicated no evidence of directional pleiotropy (P = 0.701), and Cochran's Q test showed no evidence of heterogeneity (P = 0.814) (Table 1, Fig. 2). The use of cis-SNPs as instrumental variables confirmed that there is no causal relationship between BDNF and MDD, with consistent results obtained in the second phase of analysis (Figs. 3 and 4). The results remained consistent even when SNPs associated with potential confounding factors were not excluded (Supplement Table 4 and Supplement Figure 1). |
|  | c) | If relevant, consider translating estimates of relative risk into absolute risk for a meaningful time period | NA |  |
|  | d) | Consider plots to visualize results (e.g. forest plot, scatterplot of associations between genetic variants and outcome versus between genetic variants and exposure) | 15 | Fig. 2. Causal relationship of plasma BDNF and MDD. |
| 12 | **Assessment of assumptions** |  |  |  |
|  | a) | Report the assessment of the validity of the assumptions | 4 | The MR-Egger intercept also indicated no evidence of directional pleiotropy (P = 0.701) and Cochran's Q test showed no evidence of heterogeneity (P = 0.814).To assess the reliability of our findings we performed sensitivity analyses using MR-Egger regression, the weighted median method, and the 'leave-one-out' approach |
|  | b) | Report any additional statistics (e.g., assessments of heterogeneity across genetic variants, such as *I^2^*, Q statistic or E-value) | 4 | The MR-Egger intercept also indicated no evidence of directional pleiotropy (P = 0.701) and Cochran's Q test showed no evidence of heterogeneity (P = 0.814).To assess the reliability of our findings we performed sensitivity analyses using MR-Egger regression, the weighted median method, and the 'leave-one-out' approach |
| 13 | **Sensitivity analyses and additional analyses** |  |  |  |
|  | a) | Report any sensitivity analyses to assess the robustness of the main results to violations of the assumptions | 5 | To assess the reliability of our findings we performed sensitivity analyses using MR-Egger regression, the weighted median method, and the 'leave-one-out' approach. The latter involved sequentially excluding each SNP, recalculating the pooled effect of the remaining SNPs if the IVW method showed significant results (p < 0.05) and passing heterogeneity and gene diversity tests. |
|  | b) | Report results from other sensitivity analyses or additional analyses | 5 | Our analysis included IVW, MR-Egger, weighted median, and weighted mode methods implemented via the 'TwoSampleMR' package in R (version 4.0.3) [22 23]. A causal link was considered significant with a p-value below 0.05. MR-Egger was used to address potential confounding and pleiotropy with fewer assumptions, while the weighted median approach ensured robust estimates when at least 50% of genetic variations were valid instruments. The weighting method refined estimates based on weighted analysis.  The MR-Egger intercept also indicated no evidence of directional pleiotropy (P = 0.701) and Cochran's Q test showed no evidence of heterogeneity (P = 0.814).  To assess the reliability of our findings we performed sensitivity analyses using MR-Egger regression, the weighted median method, and the 'leave-one-out' approach. |
|  | c) | Report any assessment of direction of causal relationship (e.g., bidirectional MR) | 6 | The IVW method showed no significant association between plasma BDNF levels and the risk of developing MDD (IVW odds ratio [OR] = 1.00, 95% confidence interval [CI] = 0.99–1.01, P = 0.769). Similarly, no causal effect of the BDNF gene on MDD was identified (OR = 0.91, CI = 0.23–3.56, P = 0.893). Additionally, no evidence supported a causal link between MDD and plasma BDNF levels (OR = 0.99, CI = 0.89–1.09, P = 0.783). |
|  | d) | When relevant, report and compare with estimates from non-MR analyses | NA | While previous research has suggested an association between plasma BDNF and MDD, our study did not find evidence of a causal effect of plasma BDNF on MDD risk. Furthermore, we found no evidence from Mendelian randomization indicating that genetic susceptibility to MDD affects BDNF levels |
|  | e) | Consider additional plots to visualize results (e.g., leave-one-out analyses) | 15 | Fig. 2. Causal relationship of plasma BDNF and MDD. |
|  | **DISCUSSION** |  |  |  |
| 14 | **Key results** | Summarize key results with reference to study objectives | 6 | This bidirectional MR analysis provides no evidence of a causal association between plasma BDNF levels and MDD. These findings prompt a reevaluation of plasma BDNF as a biomarker for MDD and highlight the need for further investigation into its functional role in the plasma and its levels and activity within the brain and cerebrospinal fluid. |
| 15 | **Limitations** | Discuss limitations of the study, taking into account the validity of the IV assumptions, other sources of potential bias, and imprecision. Discuss both direction and magnitude of any potential bias and any efforts to address them | 7 | This study has several limitations. Firstly, it includes only individuals of European ancestry, which may limit the generalizability of our findings to non-European populations. Further Mendelian randomization studies involving diverse ethnic groups are necessary to confirm these results across different genetic backgrounds."  Secondly, the complexity of biological systems could affect the accuracy of our findings. Since our study focused solely on plasma BDNF, it does not address the potential relationship between BDNF in cerebrospinal fluid or brain tissue and MDD. Additional research is needed to explore these associations in different biological contexts.  We employed four complementary MR methods to mitigate the risk of reverse causation bias, ensuring robust and reliable results. |
| 16 | **Interpretation** |  |  |  |
|  | a) | Meaning: Give a cautious overall interpretation of results in the context of their limitations and in comparison with other studies | 7 | While previous research has suggested an association between plasma BDNF and MDD, our study did not find evidence of a causal effect of plasma BDNF on MDD risk. Furthermore, we found no evidence from Mendelian randomization indicating that genetic susceptibility to MDD affects BDNF levels."  "These findings prompt a reevaluation of plasma BDNF as a biomarker for MDD and highlight the need for further investigation into its functional role in the plasma and its levels and activity within the brain and cerebrospinal fluid. |
|  | b) | Mechanism: Discuss underlying biological mechanisms that could drive a potential causal relationship between the investigated exposure and the outcome, and whether the gene-environment equivalence assumption is reasonable. Use causal language carefully, clarifying that IV estimates may provide causal effects only under certain assumptions | 7 | BDNF is crucial for neuronal survival, growth, and maintenance in brain circuits involved in emotional and cognitive functions. Accumulating evidence indicates that neuroplastic mechanisms mediated by BDNF are disrupted in both MDD and stress-induced animal models. Clinical and preclinical studies show that stress-related depressive pathology affects BDNF levels and function in MDD, causing disruptions in neuroplasticity at regional and circuit levels.  Most associations between BDNF and MDD have been found in brain tissue or cerebrospinal fluid. For example, the infusion of BDNF into specific brain regions such as the midbrain or hippocampus has been shown to produce antidepressant-like effects in animal models.  MR simulates a randomized controlled trial by leveraging Mendel's second law, which asserts that alleles for different genes are independently inherited during gametogenesis. Consequently, MR analyses depend on the principle that the inheritance of one trait is independent of the inheritance of others. |
|  | c) | Clinical relevance: Discuss whether the results have clinical or public policy relevance, and to what extent they inform effect sizes of possible interventions | 7 | These findings prompt a reevaluation of plasma BDNF as a biomarker for MDD and highlight the need for further investigation into its functional role in the plasma and its levels and activity within the brain and cerebrospinal fluid.  Although previous observational studies suggested an association between plasma BDNF levels and MDD, the lack of causal evidence from our MR study indicates that plasma BDNF may not be a reliable standalone biomarker for predicting or diagnosing MDD.  This suggests that interventions targeting plasma BDNF levels may not have a significant impact on MDD risk. Instead, future research should focus on understanding BDNF’s role within the brain or cerebrospinal fluid, where it might have more direct implications for neuroplasticity and MDD treatment. |
| 17 | **Generalizability** | Discuss the generalizability of the study results (a) to other populations, (b) across other exposure periods/timings, and (c) across other levels of exposure | 7 | Firstly, it includes only individuals of European ancestry, which may limit the generalizability of our findings to non-European populations. Further Mendelian randomization studies involving diverse ethnic groups are necessary to confirm these results across different genetic backgrounds.  Since our study focused solely on plasma BDNF, it does not address the potential relationship between BDNF in cerebrospinal fluid or brain tissue and MDD. Additional research is needed to explore these associations in different biological contexts. |
|  | **OTHER INFORMATION** |  |  |  |
| 18 | **Funding** | Describe sources of funding and the role of funders in the present study and, if applicable, sources of funding for the databases and original study or studies on which the present study is based | 10 | No applicable. |
| 19 | **Data and data sharing** | Provide the data used to perform all analyses or report where and how the data can be accessed, and reference these sources in the article. Provide the statistical code needed to reproduce the results in the article, or report whether the code is publicly accessible and if so, where | 10 | Data supporting the observations of this study, including the methodology, are available upon reasonable request from the corresponding authors. Publicly accessible summary statistics from GWAS were acquired from the Psychiatric Genomics Consortium (PCG) and FinnGen website. |
| 20 | **Conflicts of Interest** | All authors should declare all potential conflicts of interest | 10 | The authors declare no conflicts of interest related to this study. |

This checklist is copyrighted by the Equator Network under the Creative Commons Attribution 3.0 Unported (CC BY 3.0) license.

1. Skrivankova VW, Richmond RC, Woolf BAR, Yarmolinsky J, Davies NM, Swanson SA, et al. Strengthening the Reporting of Observational Studies in Epidemiology using Mendelian Randomization (STROBE-MR) Statement. JAMA. 2021;under review.

2. Skrivankova VW, Richmond RC, Woolf BAR, Davies NM, Swanson SA, VanderWeele TJ, et al. Strengthening the Reporting of Observational Studies in Epidemiology using Mendelian Randomisation (STROBE-MR): Explanation and Elaboration. BMJ. 2021;375:n2233.
